# Reduction in peripheral arterial stiffness after reactive hyperemia is dependent on increases in blood flow

**DOI:** 10.14814/phy2.15894

**Published:** 2023-12-18

**Authors:** Ronald E. Jackson, Natalia S. Lima, Sara R. Sherman, Philip S. Clifford

**Affiliations:** ^1^ Department of Kinesiology and Nutrition University of Illinois at Chicago Chicago Illinois USA

**Keywords:** Doppler ultrasound, pulse wave velocity, tonometry

## Abstract

The acute reduction in peripheral arterial stiffness during reactive hyperemia is assumed to be flow‐mediated; however, the mechanism remains unproven. We hypothesized that restricting the blood flow increase during reactive hyperemia would abolish the reduction in peripheral arterial stiffness. Fourteen healthy young adults (5 females, 25 ± 5 years, mean ± SD) underwent reactive hyperemia with a rapid‐release cuff on the upper arm inflated to 220 mmHg for 5 min: once with unrestricted blood flow and once with restricted blood flow by manually applying pressure to the brachial artery. Brachial–radial pulse wave velocity (PWV) was measured with tonometers over brachial and radial arteries before cuff inflation and at 5, 15, and 30 min after release. Brachial blood flow was monitored with Doppler ultrasound. Baseline brachial–radial PWV was similar between conditions (10.3 ± 1.8 vs. 10.7 ± 1.7 m/s). With unrestricted flow, PWV decreased 5 min post‐reactive hyperemia (8.6 ± 1.1 m/s; *p* < 0.05) and returned near baseline at 15 and 30 min post (*p* < 0.05). With restricted flow, PWV did not change (*p* > 0.05) post‐reactive hyperemia. Reactive hyperemia acutely reduced peripheral arterial stiffness, but not when brachial artery blood flow increase was restricted. This suggests that the reduction in peripheral arterial stiffness during reactive hyperemia depends on increased blood flow.

## 1 INTRODUCTION

Arterial stiffness is considered an important indicator of arterial wall health (Laurent et al., 2006). The stiffness of the arterial wall is influenced by a variety of factors, including changes in the composition of the arterial wall matrix (Avolio et al., 1998; Katsuda & Kaji, 2003) and properties of the vascular smooth muscle (Folkow, 1983; Heagerty et al., 1993; Rizzoni et al., 1996). Factors that influence alterations in the arterial wall matrix have an impact on the structural characteristics of the arterial wall, while factors that influence vascular smooth muscle affect contractility, which can lead to stiffening of the arterial wall. The gold standard method for in vivo assessment of arterial stiffness is the measurement of pulse wave velocity (PWV) which is the speed at which arterial pressure waves propagate through the blood vessels (Laurent et al., 2006; Pereira et al., 2015).

Recently, PWV response to a reactive hyperemia stimulus (Björnberg et al., 1990; Patterson & Whelan, 1955) has been introduced as an alternative way to evaluate endothelial function (Cauwenberghs et al., 2018; Ellins et al., 2016; Naka et al., 2006; Stoner et al., 2020). The standard approach to evaluate endothelial function is to measure the vasodilation of a conduit artery following elevation of shear stress with a reactive hyperemia stimulus (Bots et al., 2005; Thijssen et al., 2011). According to proponents of this new test, slowing of PWV during reactive hyperemia is flow‐dependent and indicative of endothelial function. Several studies have made measurements of PWV after a reactive hyperemia test in subjects who would be expected to have endothelial dysfunction. Naka et al. (2006) reported decreases in peripheral PWV after cuff occlusion in healthy individuals but not in patients with chronic heart failure. Cauwenberghs et al. (2018) reported that older subjects exhibited less decline in peripheral PWV after occlusion than younger subjects. Stoner et al. (2020) employed a procedure to induce transient endothelial dysfunction in healthy individuals by applying moderate pressure (50–70 mmHg) to the forearm using an inflatable tourniquet. They observed an increased peripheral PWV following induction of endothelial dysfunction. The papers by Naka (Naka et al., 2006), Cauewnberghs (Cauwenberghs et al., 2018), and Stoner (Stoner et al., 2020) suggest that the PWV response to reactive hyperemia depends on the activation of the conduit artery endothelium by the elevated blood flow.

Although the reduction in arterial stiffness during reactive hyperemia has been attributed to arterial flow‐mediated responses, the mechanism of action has not been proven. To do so requires manipulation of increase in conduit artery blood flow. We reasoned that attenuating the increase in blood flow by restricting arterial inflow during hyperemia would provide insight into the mechanism. We hypothesized that restricting the blood flow increase to reactive hyperemia would abolish the reduction in peripheral arterial stiffness as measured by PWV.

## 2 METHODS

### 2.1 Participants

Fourteen healthy young adults volunteered for this study. All study procedures were approved by the Institutional Review Board at the University of Illinois at Chicago (2022‐0996) and in accordance with guidelines set forth by the Declaration of Helsinki. Verbal and signed written consent were obtained from each participant before participating in this study.

Participants were excluded for smoking, presence of cardiovascular, metabolic, and pulmonary diseases. Other exclusion criteria includes hypertension or hypotension, diabetes, obesity (body mass index [BMI] >35 kg/m^2^), anti‐inflammatory medication, and pregnancy. We asked participants to refrain from exercise and caffeine for at least 24 h before their test. Participants were also asked to fast 4 h prior to their test. This study consisted of one visit in which all participants completed the experimental procedures. Height and weight were assessed using a standard stadiometer and an electronic scale, respectively.

### 2.2 Experimental procedures

Participants lay supine in a dimly lit and temperature‐controlled room for 10 min before instrumentation. Arterial beat‐by‐beat blood pressure was continuously monitored throughout the procedure using a finger cuff photoplethysmography sensor (Finometer Pro, Finapres Medical System, Amsterdam, the Netherlands) placed on the middle finger of the left hand. A rapid release cuff (Hokanson, Bellevue, WA, USA) was positioned on the right upper arm. This arm was supported on a table positioned at heart level with brachial arterial blood flow continuously monitored by Doppler ultrasound and brachial–radial PWV by pulse tonometry.

### 2.3 Pulse wave velocity

Two pulse tonometers (Millar Instruments, Houston, TX, USA) were used to obtain arterial pressure waveforms between the brachial artery and radial artery of the right arm. The arterial pressure waveform signals were recorded in real time and stored at 1000 Hz using Powerlab (AD instruments, Colorado Springs, CO, USA). PWV was calculated from the distance between the brachial and radial arterial sites and the pulse transit time between arterial pressure waves. The distance between the placement of the pulse tonometer on the brachial and radial artery was measured as a straight line with a tape measure. Pulse transit time was determined by the time delay between the foot of the brachial and radial arterial pulse waves. At least seven consecutive brachial and radial pulse waves were averaged. A macro written in Powerlab was used to automate the calculations. The coefficient of variation for resting brachial–radial PWV is 1.7% in our laboratory.

### 2.4 Brachial artery blood flow

Brachial artery blood flow in the experimental arm was measured by high‐resolution Doppler ultrasound (Prosound Alpha 7, Hitachi‐Aloka, Japan) with a linear probe operating between 5 and 13 MHz and an insonation angle at 60^ο^. Mean blood velocity was analyzed using blood velocity analysis software (Cardiovascular Suite, Quipu, Pisa, Italy). Brachial artery blood flow (mL/min) was calculated as follows: ([conduit artery diameter (cm)/2]^2^ × π × mean blood velocity (cm/s) × 60). Blood flow measurements were calculated as 1‐s averages. The peak blood flow response was established by identifying the highest blood flow value after releasing of the cuff occlusion. The area under the curve (AUC) was determined starting from the cuff release and continuing for 3 min. AUC was determined using GraphPad Prism version 9.0.0 (GraphPad Software, San Diego, CA, USA, www.graphpad.com).

### 2.5 Reactive hyperemia

Participants underwent two reactive hyperemia challenges on the same day: one with unrestricted blood flow and one with restricted blood flow, the order of which was counterbalanced. After the conclusion of each reactive hyperemia challenge, there was a 10‐min interval before the initiation of the subsequent challenge. To perform reactive hyperemia challenges, the rapid‐release cuff on the upper right arm was inflated to 220 mmHg to occlude arterial inflow and sustained for 5 min. Brachial arterial blood flow was continuously monitored from 1 min before cuff inflation (e.g., baseline) to 3 min after release. PWV measurements were made before cuff inflation (e.g., baseline) and 5, 15, and 30 min after release of the cuff pressure. For the blood flow restriction trial, the investigator manually applied pressure with the thumb to the brachial artery at the time of cuff release to restrict the increase in blood flow in the brachial artery for the subsequent 3 min (Blair et al., 1959; Messere et al., 2018; Pyke et al., 2008). To ensure that adequate pressure was applied to restrict the increase in blood flow, brachial arterial blood flow was continuously monitored by Doppler ultrasound.

### 2.6 Statistical analysis

Statistical analyses were performed using IBM SPSS version 28.0. (IBM Corp., Armonk, NY, USA). Statistical significance was a priori defined at *p* ≤ 0.05. Repeated measures ANOVA was used to evaluate differences in PWV between reactive hyperemia with unrestricted and restricted blood flow. When a significant effect was verified, post hoc analysis using Bonferroni adjustment was applied. Paired sample *t*‐tests were performed to evaluate the differences in peak blood flow and AUC between reactive hyperemia with unrestricted and restricted blood flow. Data were presented as mean ± standard deviation.

The sample size for this study was calculated based on the variability in the study by Naka et al. (2006). The results of the power analysis indicated that 10 participants would allow detection of a meaningful difference of 1 m/s in PWV with >80% power and an alpha at 0.05.

## 3 RESULTS

A total of 14 participants were included in this study (5 females, 9 males) and descriptive characteristics are presented in Table 1.

**TABLE 1:** Descriptive characteristics of participants.

	Group (*n* = 14)
Age, years	25 ± 5
Male/Female, *n*	9/5
Height, cm	171.4 ± 10
Weight, kg	71.9 ± 15
BMI, kg/m^2^	24.7 ± 4

*Note*: Data are presented as mean ± SD.

Abbreviation: BMI, body mass index.

**FIGURE 1:**
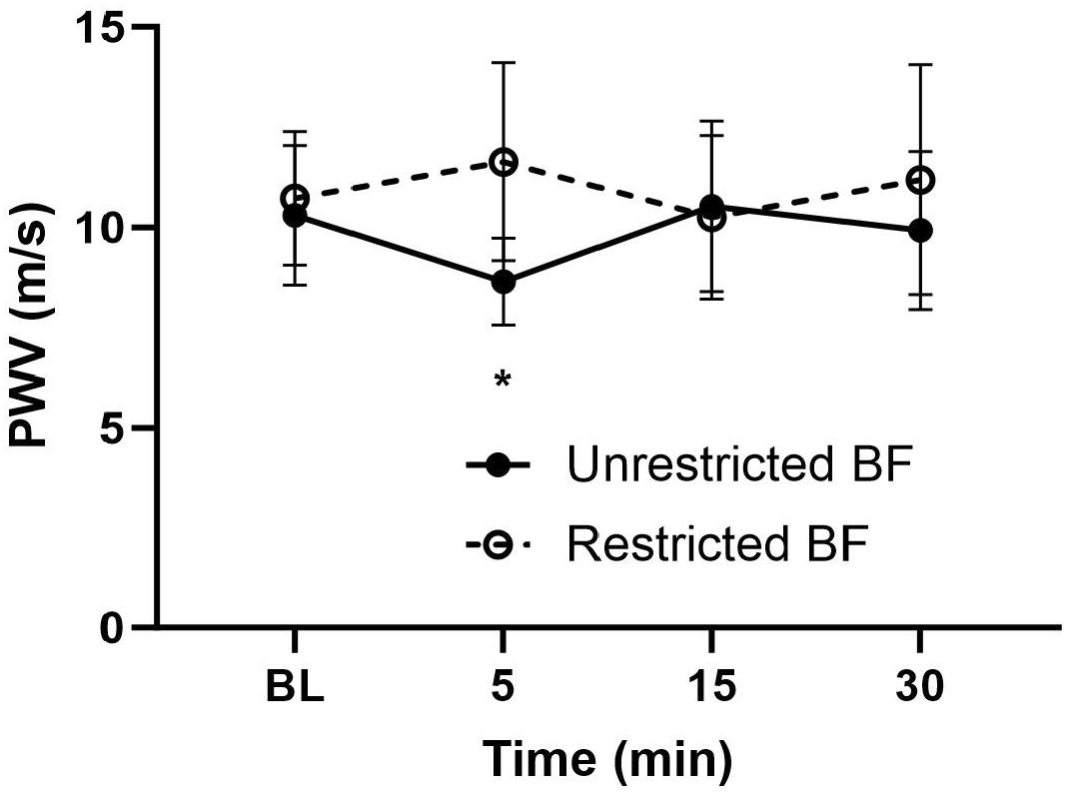
Brachial to radial pulse wave velocity (PWV) measurements in the experimental arm following reactive hyperemia with unrestricted and restricted blood flow. BL = baseline *Significant differences between unrestricted and restricted blood flow (*p* < 0.001), *n* = 14.

Figure 1 depicts the brachial to radial PWV measurements following reactive hyperemia with unrestricted and restricted blood flow. Brachial–radial PWV was similar between conditions at baseline (10.3 ± 1.8 vs. 10.7 ± 1.7 m/s). With unrestricted blood flow, brachial–radial PWV decreased by ~17% 5‐min post reactive hyperemia (8.6 ± 1.1 m/s; *p* = 0.016) compared to baseline but not lower than baseline at 15 and 30 min post (*p* < 0.05). With restricted blood flow, brachial–radial PWV did not change significantly (*p* > 0.05) from baseline in response to reactive hyperemia. There was a significant time × condition interaction between the two reactive hyperemia challenges (*p* < 0.001). Mean arterial pressure (MAP) was not significantly different (*p* > 0.05) across time points (Table 2).

**TABLE 2:** Mean arterial pressure (mmHg) across time points.

	Baseline	5 min	15 min	30 min
Unrestricted blood flow	90 ± 10	92 ± 9	91 ± 8	93 ± 9
Restricted blood flow	92 ± 10	92 ± 8	92 ± 11	91 ± 10

*Note*: Data are presented as mean ± SD.

**FIGURE 2:**
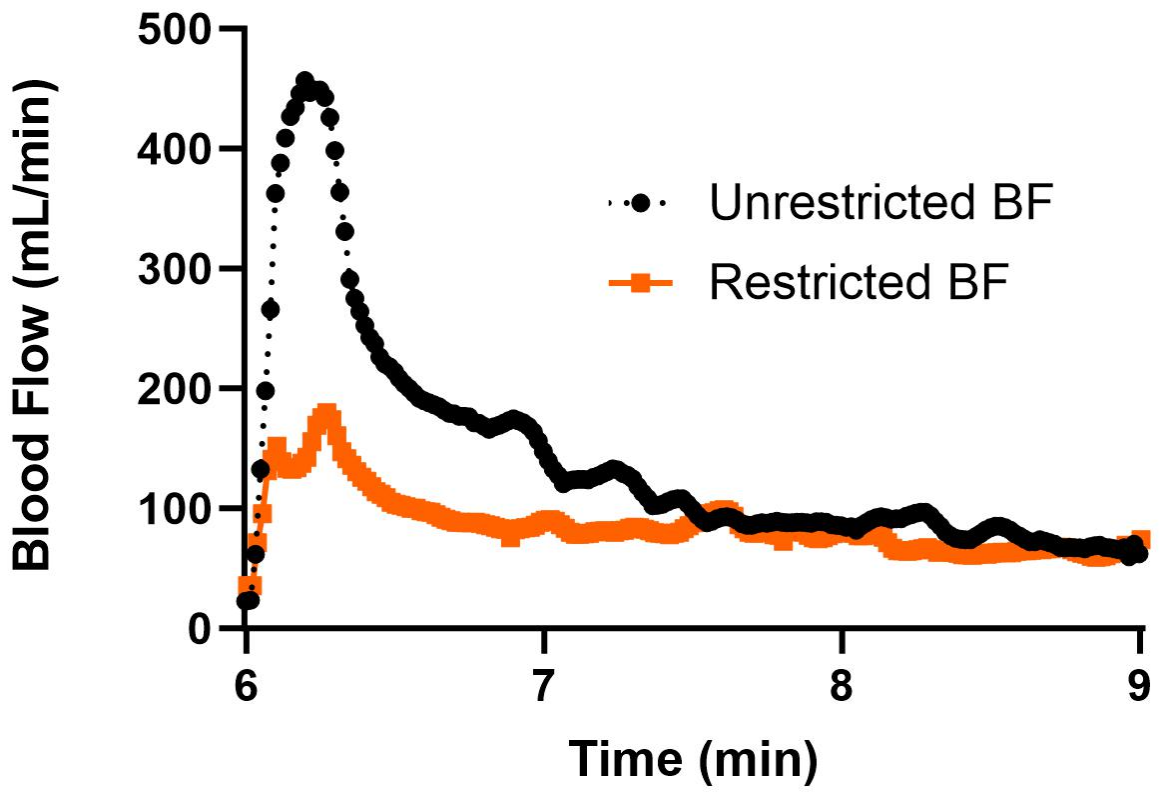
Ensemble average of individual participants' brachial blood flow responses to reactive hyperemia with unrestricted and restricted blood flow. *n* = 14.

The blood flow responses to the two reactive hyperemia challenges are shown in Figure 2. As expected, blood flow responses were greater following reactive hyperemia with unrestricted blood flow than when blood flow was restricted.

**FIGURE 3:**
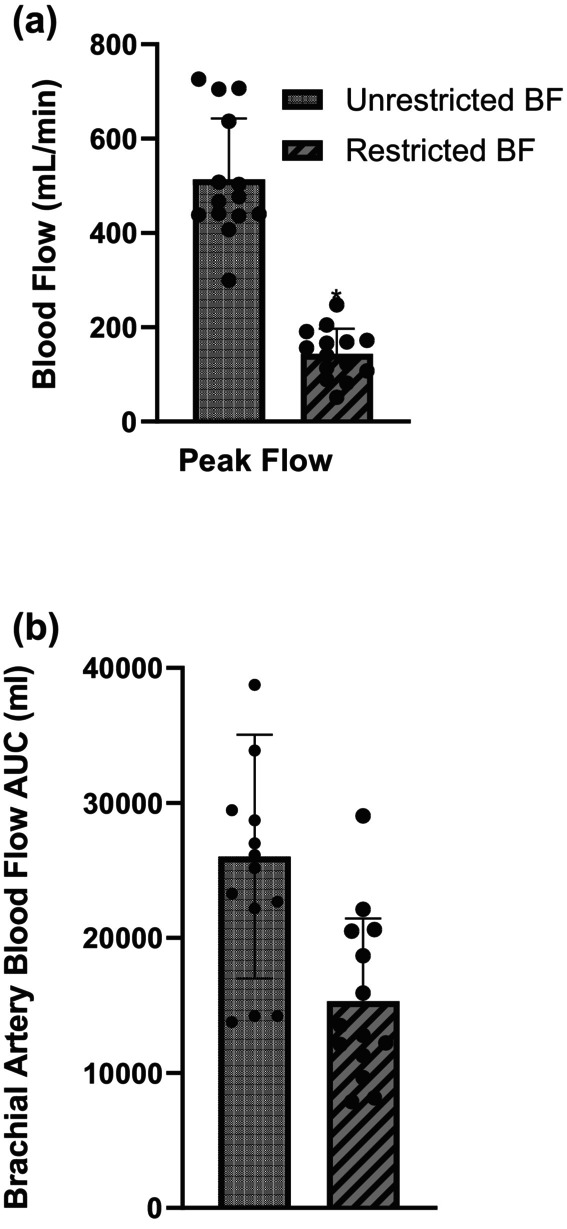
Peak brachial blood flow (a) responses when blood flow to the experimental arm was unrestricted versus restricted following reactive hyperemia. Brachial blood flow area under the curve (AUC, b) when blood flow to the experimental arm was unrestricted versus restricted following reactive hyperemia. *Significant difference between unrestricted and restricted blood flow (*p* < 0.001). *n* = 14.

Figure 3a shows peak blood flow and in Figure 3b AUC following reactive hyperemia. When blood flow was restricted following reactive hyperemia, both peak brachial artery blood flow (513 ± 129 vs. 144 ± 53 mL/min; *p* < 0.001) and AUC (26,022 ± 9038 vs. 15,317 ± 6110 mL; *p* < 0.001) were significantly reduced.

## 4 DISCUSSION

In this study, two reactive hyperemia challenges were conducted with the objective of assessing how increases in blood flow influence peripheral arterial stiffness. A distinctive aspect of this investigation was manipulating the blood flow response to reactive hyperemia via blood flow restriction. The main outcomes of the study are as follows. First, we observed an acute reduction in peripheral arterial stiffness following reactive hyperemia without any blood flow restriction. Second, restricting blood flow to curtail the hyperemic response abolished the reduction in peripheral arterial stiffness. Our results imply that the reduction in peripheral arterial stiffness following reactive hyperemia is due to the increase in blood flow.

In the present study, conducting reactive hyperemia without restricting blood flow resulted in a reduction in peripheral arterial stiffness of ~17% at 5 min. However, this reduction in peripheral arterial stiffness was transient, returning to baseline levels at 15 and 30 min. Comparable outcomes have been observed in prior research regarding changes in peripheral arterial stiffness to a reactive hyperemia challenge. Naka et al. (2006) demonstrated that among normal healthy individuals, an acute reduction of 14% in PWV during reactive hyperemia of the upper limbs, with PWV levels returning to baseline within 10 min. Cauwenberghs et al. (2018) reported a peak reduction of ~17% in brachial–radial PWV following a 5‐min occlusion. Reactive hyperemia elicited an 18% reduction in brachial–radial PWV in healthy controls studied by Ellins et al. (2016). Thus, it is a consistent finding that reactive hyperemia acutely reduces peripheral arterial stiffness.

The reduction in peripheral arterial stiffness during reactive hyperemia has been attributed to a flow‐mediated mechanism (Cauwenberghs et al., 2018; Naka et al., 2006; Stoner et al., 2020). The concept is that reactive hyperemia initiates an acute phase of augmented blood flow, leading to an elevation in shear stress which stimulates endothelial nitric oxide (NO) release. This elevated NO release results in conduit artery vasodilation and increases arterial diameter. The results of several studies support this schema. Infusion of an NO synthase inhibitor (L‐NMMA) increased baseline PWV in healthy subjects (Naka et al., 2006). Patients with chronic endothelial dysfunction, that is familial hypercholesterolemia (Ellins et al., 2016) and congestive heart failure (Cauwenberghs et al., 2018), have an attenuated reduction in PWV to reactive hyperemia. After inducing acute endothelial dysfunction in healthy subjects (Stoner et al., 2020), there was an attenuated reduction in brachial–radial PWV to reactive hyperemia. In all the above studies, flow‐mediated dilation induced during reactive hyperemia is suggested as the mechanism for the reduction in peripheral arterial stiffness. However, they did not assess conduit artery blood flow. To provide mechanistic insight of how increases in blood flow influence peripheral arterial stiffness, we both measured and manipulated brachial artery blood flow following reactive hyperemia. The results indicate that the restriction of blood flow, causing a diminished hyperemic response following reactive hyperemia, abolished the reduction in peripheral arterial stiffness. This suggests that the reduction in peripheral arterial stiffness following reactive hyperemia depends on increases in blood flow.

Although there is a myogenic component to reactive hyperemia, it is traditionally explained by the metabolic stimulus arising from the accumulation of metabolites in the ischemic tissue (Björnberg et al., 1990; Jasperse et al., 2015). Given that both reactive hyperemia challenges in this study (i.e., unrestricted and restricted blood flow) involved identical occlusion durations, it is reasonable to infer that the accumulation of metabolites was similar, indicating that the metabolic response was consistent. Thus, the primary difference that sets them apart is blood flow. The results of this study show that when blood flow was unrestricted, resulting in a more pronounced hyperemic response, there was an acute reduction in peripheral arterial stiffness. This contrasted to the situation when blood flow was restricted, and peripheral arterial stiffness remained largely unchanged. The inference is that it is the increase in blood flow, rather than the metabolic stimulus, that drives the observed acute reductions in peripheral arterial stiffness. In both reactive hyperemia challenges, no change in systemic arterial pressure was detected, indicating that blood pressure was not a contributing factor to our findings.

It is interesting to consider the implications of the current results in relation to other stimuli that induce acute increases in blood flow such as exercise, heat, and mechanical compression. Peripheral arterial stiffness has been shown to be reduced acutely by exercise (Ranadive et al., 2012; Sugawara et al., 2003), heat (Cheng et al., 2021), and passive mechanical compression (Heffernan et al., 2007). Given the commonality that reactive hyperemia shares with these stimuli, it appears that any maneuver that elicits increases in conduit artery blood flow will affect peripheral arterial stiffness.

This study adds to the existing body of knowledge regarding the PWV response to reactive hyperemia. The strengths of this study include employing applanation tonometry to measure PWV, a method widely recognized as the gold standard for PWV measurement. There are some limitations to this study. First, the manual pressure applied to the brachial artery was not standardized across all participants but was performed by the same research technician on all 14 participants. Additionally, the subjects were predominantly young adults. Age is a factor that alters arterial stiffness. There is a need for future investigations to specifically address potential disparities in PWV following reactive hyperemia associated with varying age groups.

## 5 CONCLUSION

In summary, the results show that (1) reactive hyperemia with unrestricted blood flow leads to an acute reduction in peripheral arterial stiffness, and (2) this reduction in peripheral arterial stiffness is abolished when increases in brachial blood flow during reactive hyperemia are restricted. These results underscore the critical role of blood flow in influencing peripheral arterial stiffness measured with brachial–radial PWV. Therefore, the acute reduction in peripheral stiffness following reactive hyperemia is primarily driven by the mechanism of increased blood flow.

## AUTHOR CONTRIBUTIONS

Ronald E. Jackson conceived and designed research, performed experiments, analyzed data, interpreted results of experiments, prepared figures, drafted manuscript, edited and revised the manuscript, and approved the final version of manuscript. Natalia S. Lima conceived and designed research, performed experiments, interpreted results of experiments, edited and revised the manuscript, and approved final version of manuscript. Sara R. Sherman performed experiments, edited and revised the manuscript, and approved the final version of the manuscript. Philip S. Clifford conceived and designed research, interpreted results of experiments, edited and revised the manuscript, and approved the final version of the manuscript.

## FUNDING INFORMATION

No external funding was received for this research. REJ was supported by the National Heart, Lung, and Blood Institute (T32HL13463404).

## CONFLICT OF INTEREST STATEMENT

The authors declare no conflicts of interest.

## ETHICS STATEMENT

All study procedures were approved by the Institutional Review Board at the University of Illinois at Chicago (2022‐0996) and in accordance with guidelines set forth by the Declaration of Helsinki. Verbal and signed written consent were obtained from each participant before participating in this study.

## Data Availability

This dataset is available from INDIGO 10.25417/uic.24226165.
